# NEDD9 Inhibition by miR-25-5p Activation Is Critically Involved in Co-Treatment of Melatonin- and Pterostilbene-Induced Apoptosis in Colorectal Cancer Cells

**DOI:** 10.3390/cancers11111684

**Published:** 2019-10-29

**Authors:** Ji Hoon Jung, Eun Ah Shin, Ju-Ha Kim, Deok Yong Sim, Hyemin Lee, Ji Eon Park, Hyo-Jung Lee, Sung-Hoon Kim

**Affiliations:** Cancer Molecular Targeted Herbal Research Laboratory, College of Kyung Hee Medicine, Kyung Hee University, Seoul 02447, Koreaeunah1008@khu.ac.kr (E.A.S.); 964juha@daum.net (J.-H.K.); simdy0821@naver.com (D.Y.S.); glansy555@gmail.com (H.L.); wdnk77@naver.com (J.E.P.); hyonice77@naver.com (H.-J.L.)

**Keywords:** MLT, Ptero, synergy, apoptosis, miR-25-5p, NEDD9

## Abstract

The underlying interaction between melatonin (MLT) and daily fruit intake still remains unclear to date, despite multibiological effects of MLT. Herein, the apoptotic mechanism by co-treatment of MLT and pterostilbene (Ptero) contained mainly in grape and blueberries was elucidated in colorectal cancers (CRCs). MLT and Ptero co-treatment (MLT+Ptero) showed synergistic cytotoxicity compared with MLT or Ptero alone, reduced the number of colonies and Ki67 expression, and also increased terminal deoxynucleotidyl transferase dUTP nick end labeling- (TUNEL) positive cells and reactive oxygen species (ROS) production in CRCs. Consistently, MLT+Ptero cleaved caspase 3 and poly (ADP-ribose) polymerase (PARP), activated sex-determining region Y-Box10 (SOX10), and also attenuated the expression of Bcl-xL, neural precursor cell expressed developmentally downregulated protein 9 (NEDD9), and SOX9 in CRCs. Additionally, MLT+Ptero induced differentially expressed microRNAs (upregulation: miR-25-5p, miR-542-5p, miR-711, miR-4725-3p, and miR-4484; downregulation: miR-4504, miR-668-3p, miR-3121-5p, miR-195-3p, and miR-5194) in HT29 cells. Consistently, MLT +Ptero upregulated miR-25-5p at mRNA level and conversely NEDD9 overexpression or miR-25-5p inhibitor reversed the ability of MLT+Ptero to increase cytotoxicity, suppress colony formation, and cleave PARP in CRCs. Furthermore, immunofluorescence confirmed miR-25-5p inhibitor reversed the reduced fluorescence of NEDD9 and increased SOX10 by MLT+Ptero in HT29 cells. Taken together, our findings provided evidence that MLT+Ptero enhances apoptosis via miR-25-5p mediated NEDD9 inhibition in colon cancer cells as a potent strategy for colorectal cancer therapy.

## 1. Introduction

Though melatonin (MLT), or N-acetyl-5-methoxytryptamine produced from the pineal gland [1,2] and other cells/organs [3], was known to have antitumor effect through multitarget pathway at a wide range of physiological concentrations in several cancers such as breast [4,5,6], colon [7,8], liver [9], and prostate [10,11,12] cancers, recently, combination therapy of MLT with anticancer agents including paclitaxel [13], cisplatin [14], 5-fluorouracil (5-FU) [15], and sorafenib [9], or phytochemicals such as flavone [7], ghrelin [16] and ursolic acid [17], is in the spotlight.

Stilbenes, including pterostilbene (3′,5′-dimethoxy-4E-stilbenol; Ptero) and resveratrol are a class of natural polyphenolic compounds contained in several fruits, such as grape leaves, vines, blueberries, almonds, and various Vaccinium berries [18]. Especially Ptero, contained in blueberries, grape leaves and vines, almonds, and Pterocarpus marsupium heartwood, was known to exert an antitumor effect via antiangiogenic [19], antimetastatic [20], and apoptotic [21] activities. Notably, Ptero exhibited an antitumor effect better than resveratrol in colon [22] and cervical [23] cancers.

Colorectal cancer (CRC) is reported to be the third most common type of cancers and the second leading cause of cancer-related deaths all over the world [24]. Nevertheless, the incidence of colon cancer has been increasing along with obesity worldwide [25], and, recently, molecular target therapy including microRNAs (miRNAs) has been highlighted in cancer therapy [26,27].

MicroRNAs [28], a class of small noncoding RNAs [29], play pivotal roles in apoptosis induction as a tumor suppressor or an oncogene via inhibition of degradation or translation of target molecules [30]. Recent studies have revealed that some miRNAs are critically involved in MLT-induced apoptosis in several cancers, such as breast [31] and colon cancers [32]. Among them, though miR-25 was known to promote proliferation in several cancers, such as lung cancer [33], prostate cancer [34], ovarian cancer [35], and colorectal cancer [36] as an oncogene, miR-25-5p was reported to suppress PKCζ as a tumor suppressor [37,38]. Also, among several target molecules, neural precursor cell expressed developmentally downregulated 9 (NEDD9), so called Cas-L (Crk-associated substrate L) or human enhancer of filamentation1 (HEF1), promotes proliferation [39] and metastasis [19,39] in breast cancer, colorectal cancer, and head and neck cancer [40,41]. Of note, Cheung and his colleagues reported at the 2014 International SOX Research Conference that the pro-survival function of NEDD9 was mediated by SOX9 or its negative molecule SOX10. Thus, in the current work, the underlying apoptotic mechanism by MLT+Ptero was examined in HT29, SW480, and HCT116 cells in association with miR-25-5p mediated NEDD9 signaling.

## 2. Results

### 2.1. MLT+Ptero Exerted Synergistic Cytotoxicity in CRCs 

**Figure 1:**
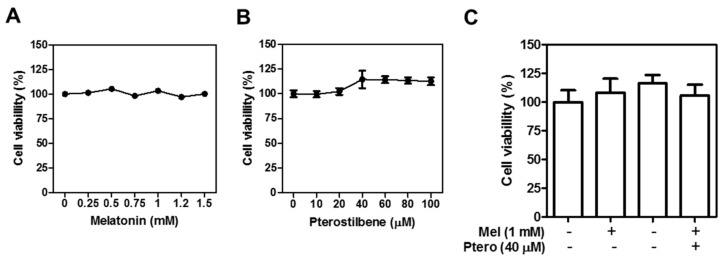

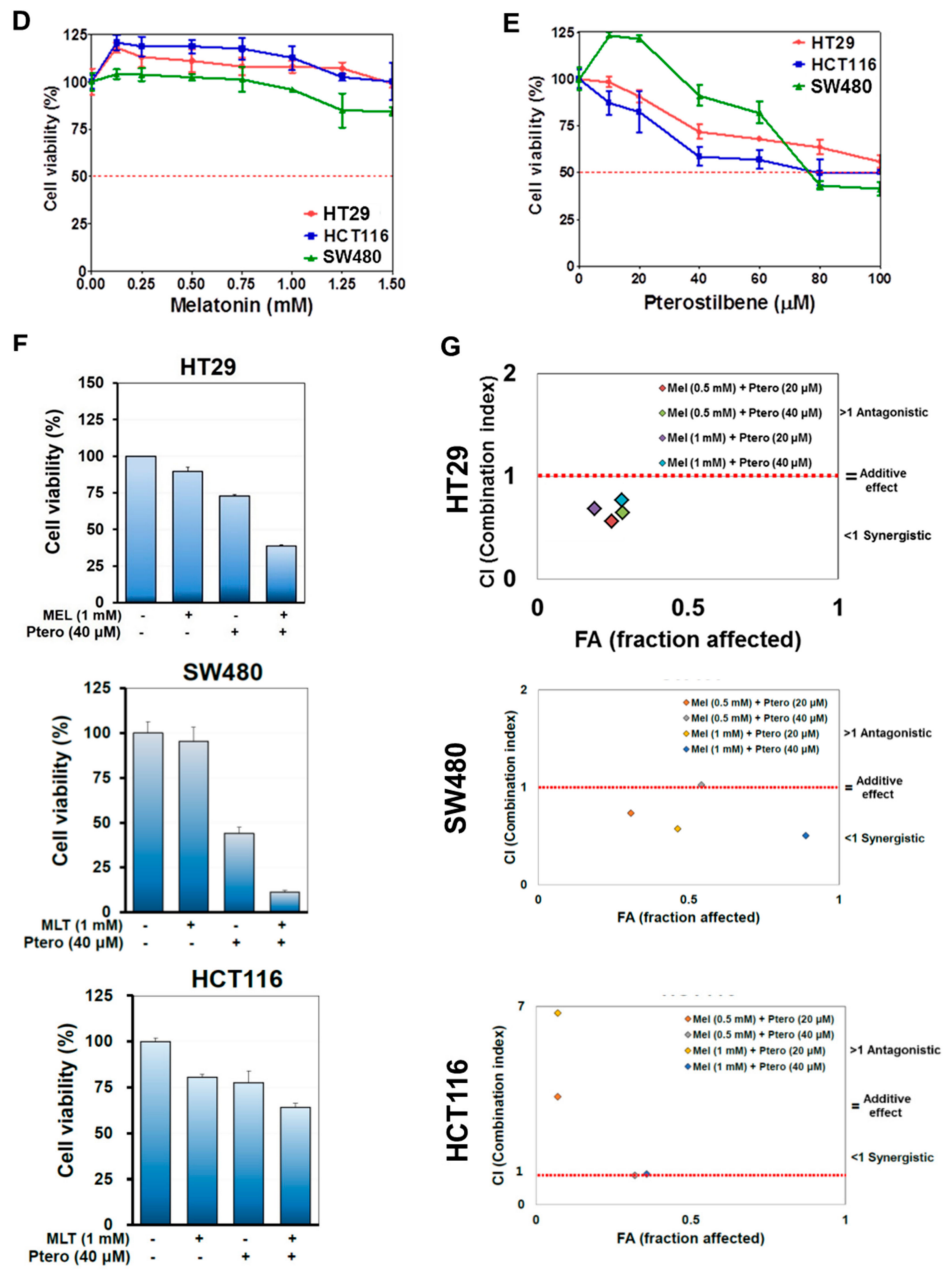
Synergistic cytotoxicity of melatonin (MLT) and pterostilbene (Ptero) co-treatment (MLT+Ptero) in colon cancer cells. Cytotoxicity of MLT (**A**), Ptero (**B**), MLT with or without Ptero (**C**) in CCD-18Co. Cytotoxicity of MLT (**D**) or Ptero (**E**), HT29, SW480, and HCT116 cells by MTT assay. Three colon cancer cell lines were exposed to the indicated concentrations of MLT or Ptero for 24 h and then cell viability was analyzed by MTT assay. (**F**) Bar graphs indicate cytotoxicity of MLT and/or Ptero in three colon cancer cells by MTT assay. Data represent means ± SD from three separate experiments. (**G**) Combination index (CI) for cytotoxicity of MLT and Ptero was determined by Chou-Talalay method using the Calcusyn software.

Cytotoxicity of MLT and/or Ptero was examined in CCD18-Co, HCT116, SW480, and HT29 cells by using 3-(4,5-dimethylthiazol-2-yl)-2,5-diphenyltetrazolium bromide (MTT) assay. Interestingly, MLT and/or Ptero do not affect the viability of normal colon CCD18-Co cells (Figure 1A–C). Ptero significantly reduced the viability of HT29, SW480, and HCT116 cells compared to MLT alone (Figure 1D,E). To assess the synergistic cytotoxicity of MLT+Ptero, three colon cancer cells were exposed to Ptero (40 µM) and MLT (1 mM) for 24 h. MLT+Ptero exhibited significant cytotoxicity in three colon cancer cells (Figure 1F). The cytotoxic synergy of MLT+Ptero was also confirmed in HT29, SW480, and HCT116 cells by the Chou and Talalay method, since combination index (CI) values for cytotoxicity of MLT+Ptero were below 1 at all fraction-affected (Fa) points (Figure 1G).

### 2.2. MLT+Ptero Cleaved PARP and Caspase-3 and Modulated NEDD9-Related Proteins in CRCs

**Figure 2:**
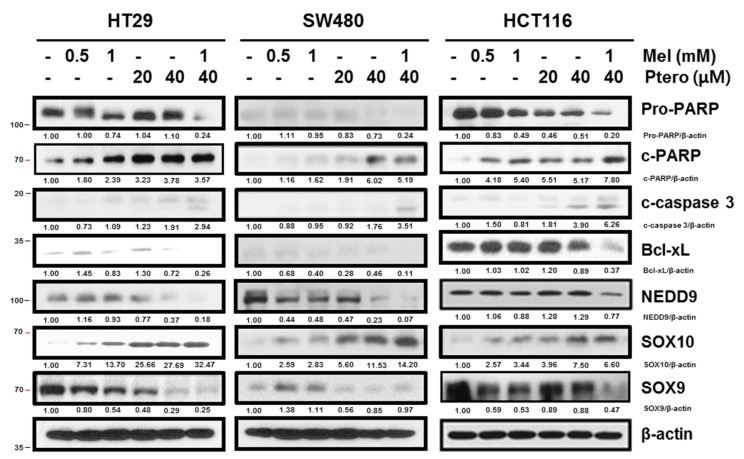
Effect of MLT+Ptero on apoptosis-related proteins in colon cancer cells. HCT116, SW480, and HT29 colon cancer cells were exposed to MLT (0.5, 1 mM) and/or Ptero (20, 40 µM) for 24 h and then were subjected to Western blotting for PARP, cleaved caspse-3, Bcl-xL, NEDD9, SOX10, SOX9, and β-actin.

Generally, apoptosis is induced via mitochondrial-dependent intrinsic pathway or cell death extrinsic pathway [42] Herein, co-treatment of Ptero (20, 40 µM) and MLT (0.5, 1 mM) Poly (ADP-ribose) polymerase (PARP) and caspase-3 in CRCs by Western blotting. Also, co-treatment of MLT (0.5, 1 mM) and Ptero (20, 40 µM) suppressed the expression of NEDD9, SOX9, and Bcl-xL, and upregulated SOX10 in CRCs (Figure 2). Given that overexpression of NEDD9 implies poor prognosis in colorectal cancer patients [43], we confirmed a dramatic effect of MLT (0.5, 1 mM) and/or Ptero (20, 40 µM) on NEDD and SOX10/9, especially in SW480 and HT29 CRCs better than in HCT116 cells.

### 2.3. MLT+Ptero Increased the Number of TUNEL-Positive Cells and ROS Production and Suppressed Ki67-Positive Cells in CRCs 

**Figure 3:**
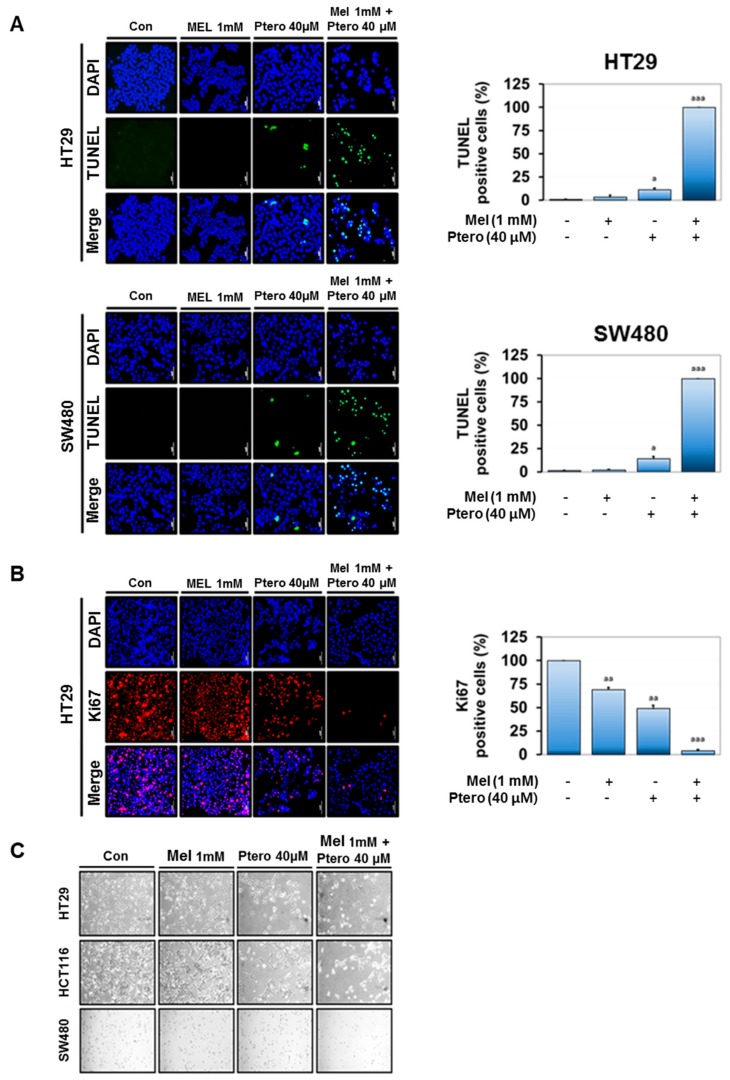

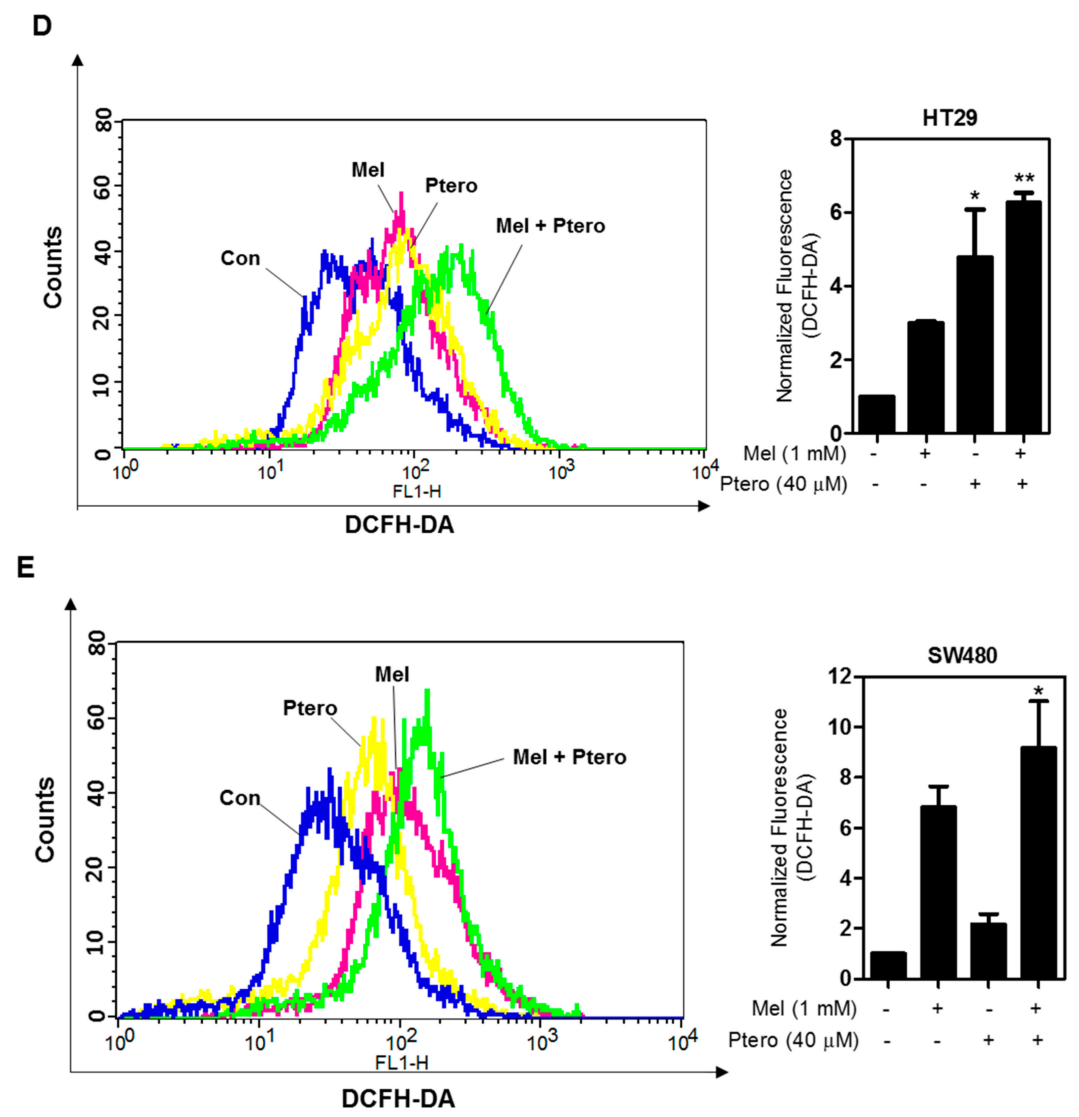
Effect of MLT+Ptero on TUNEL-positive cells, Ki67 expression, and reactive oxygen species (ROS) production in colon cancer cells. (**A**) Effect of MLT+Ptero on TUNEL-positive cells in HT29 and SW480 cells. HT29 and SW480 cells were exposed to MLT (1 mM) and/or Ptero (40 µM) for TUNEL staining. The fluorescent signals from fragmented DNA (green) and 4′,6-diamidino-2-phenylindole. (DAPI) (blue) were visualized and photographed by a FLUOVIEW FV10i confocal microscopy. Magnification bar = 50 µm. Bar graphs represent quantification of TUNEL-positive cells (%). Data represent means ± SEM of triplicate samples. * *p* < 0.05, *** *p* < 0.001 vs. untreated control. (**B**) Effect of MLT+Ptero on Ki67 expression in HT29 cells. Immunofluorescence staining of proliferation marker Ki67 in HT29 cells. Nuclei were stained by DAPI (blue) stain and anti-rabbit Alexa Fluor 546 (red). ** *p* < 0.01, *** *p* < 0.001 vs. untreated control by one-way ANOVA test. (**C**) Effect of MLT+Ptero on apoptotic morphological changes in HT29, SW480, and HCT116 cells. Following exposure to MLT and/or Ptero in three colon cancer cells for 24 h, apoptotic morphology of the cells was observed in the cells under phase contrast microscope. (**D**,**E**) Effect of MLT+Ptero on ROS production in HT29 or SW480 cells. HT29 or SW480 cells were treated with MLT (1 mM) and/or Ptero (40 μM) for 24 h and then 10 μM Dichloro-dihydro-fluorescein diacetate (DCFH-DA) for 30 min at 37 °C. Fluorescence intensity was measured by Dichloro-dihydro-fluorescein diacetate (FACS) Calibur. Bar graphs showed quantification of ROS generation. Data represent means ± SD. * *p* < 0.05 versus untreated control (*n* = 2, one-way ANOVA, Tukey’s test).

To confirm whether the cytotoxicity of MLT+Ptero was induced by apoptosis, Terminal deoxynucleotidyl transferase dUTP nick end labeling (TUNEL) assay was conducted in HT29 and SW480 cells, since TUNEL-positive staining implies a feature of apoptosis. The numbers of TUNEL-positive cells were significantly increased by MLT+Ptero in HT29 and SW480 cells compared to MLT or Ptero alone (Figure 3A). Furthermore, MLT+Ptero attenuated the expression of Ki67 as a biomarker of cell proliferation in HT29 cells compared to the untreated control (Figure 3B). Consistently, the apoptotic features, such as apoptotic bodies and cell shrinkages, were observed in Ptero- (40 μM) and MLT- (1mM) treated three CRCs (Figure 3C). Also, MLT+Ptero significantly increased reactive oxygen species (ROS) production in HT29 or SW480 cells compared to MLT or Ptero alone (Figure 3D,E).

### 2.4. Differentially Expressed microRNA Profile in MLT- and/or Ptero-Treated HT29 Cells 

**Figure 4:**
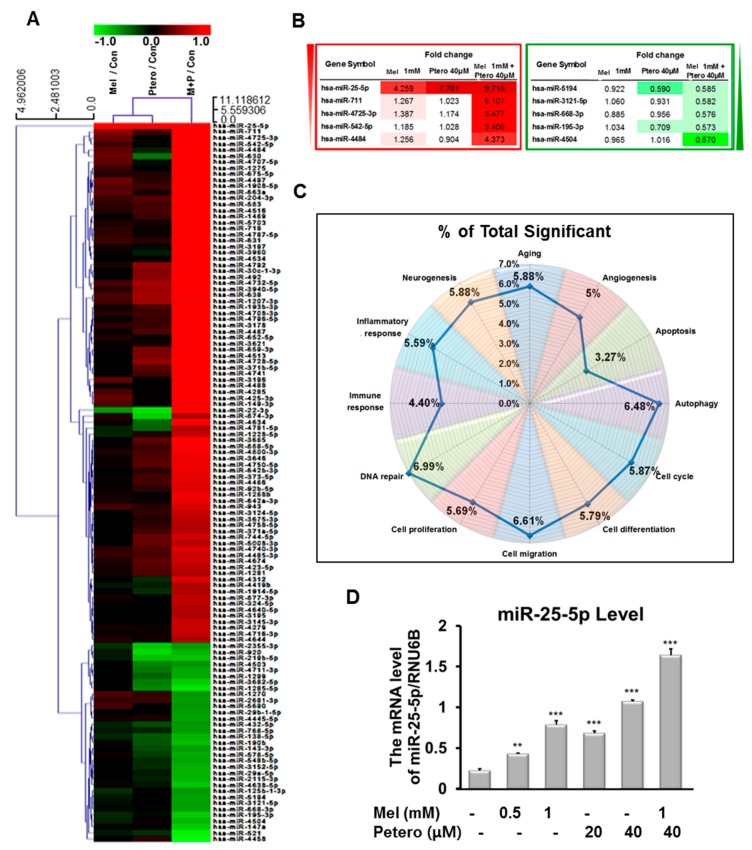
Differential profiles of microRNAs in HT29 cells treated with MLT and/or Ptero by microarray analysis. (**A**) Heat map and summary of microRNAs (miRNAs) enriched in MLT- (1 mM) and/or Ptero- (40 µM) treated HT29 cells. Red and green indicate upregulation and downregulation of miRNAs, respectively. (**B**) The miRNAs differentially upregulated by over 2 folds or downregulated by below 0.5 fold by MLT and Ptero co-treatment in HT29 cells. (**C**) Kyoto Encyclopedia of Genes and Genomes (KEGG) signaling pathways in MLT- and/or Ptero-treated HT29 cells. (**D**) Effect of MLT and/or Ptero treatment on miR-25-5p in HT29 cells by quantitative RT-PCR (qRT-PCR) analysis.

To confirm whether MLT and/or Ptero treatment modulated the miRNA signature, microarray analysis was conducted in Ptero- and/or MLT-treated HT29 cells. The heat map showed a differentially expressed miRNA profile in Ptero- and/or MLT-treated HT29 cells (Figure 4A and Table S1). Furthermore, MLT+Ptero induced differential upregulation of miR-25-5P, miR-4725-3P, miR-542-5P, miR-711, and miR-4484, and downregulation of miR-668-3p, miR-4504, miR-195-3p, miR-3121-5p, and miR-5194 in HT29 cells (Figure 4B). Also, Kyoto Encyclopedia of Genes and Genomes (KEGG) pathway analysis showed that MLT+Ptero regulated several signaling pathways on DNA repair (6.99%), autophagy (6.48%), cell migration (6.61%), aging (5.88%), neurogenesis (5.88%), cell cycle (5.87%), cell differentiation (5.79%), cell proliferation (5.69%), inflammatory response (5.59%), angiogenesis (5%), immune response (4.40%), and apoptosis (3.27%) in HT29 cells (Figure 4C). Consistent with microarray data, miR-25-5p upregulation by MLT+Ptero was verified in a concentration-dependent fashion in HT29 cells by quantitative RT-PCR (qRT-PCR) analysis (Figure 4D).

### 2.5. Late-Stage Autophagy Inhibitor CQ, but not 3-MA, Enhanced Cytotoxicity and Decreased p62 and Activated LC3II in Atorvastatin-Treated H596, H460, and H1299 Cells

**Figure 5:**
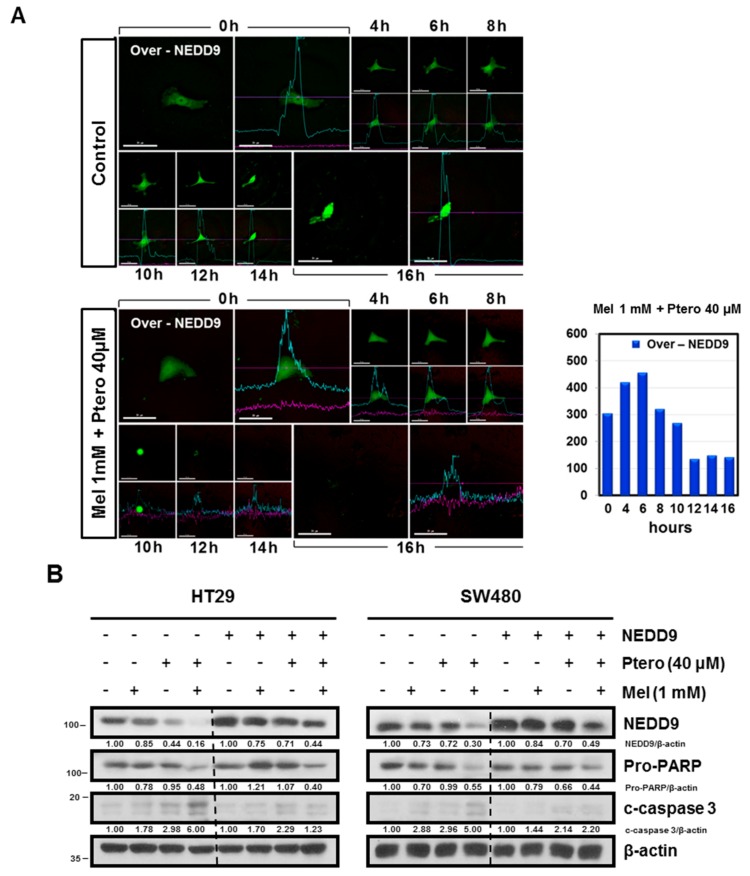

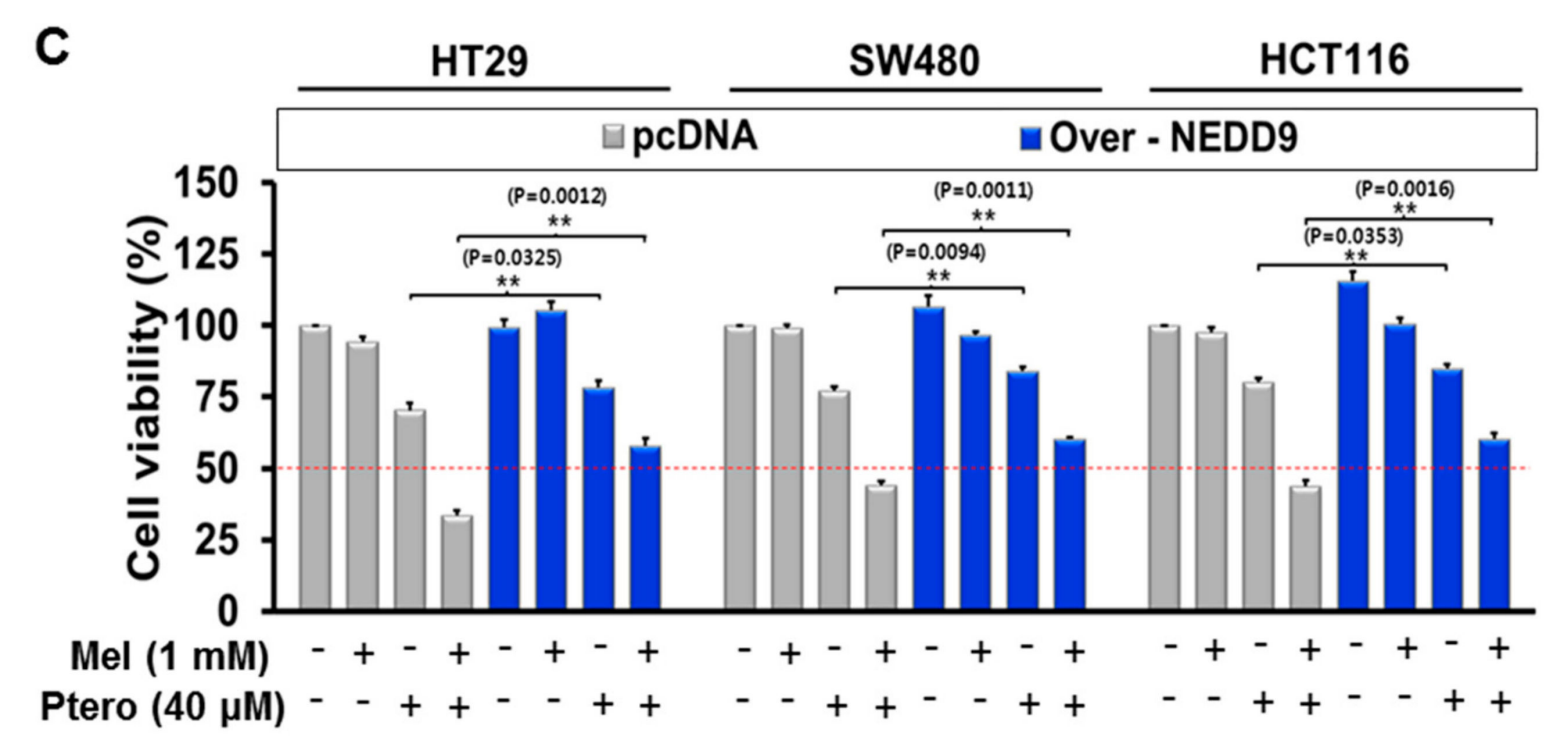
NEDD9 overexpression reversed the ability of MLT+Ptero to exert cytotoxicity, reduce NEDD9 expression, and induce cleavage of PARP and caspase 3 in colon cancer cells. (**A**) MLT+Ptero completely reduced fluorescence of GFP NEDD9 in HT29 cells transfected with NEDD overexpression plasmid. Stable expression of GFP NEDD was observed in HT29 cells transfected with NEDD9 overexpression plasmid under inverted fluorescence. Time-lapse microscopy images are the maximum intensity with projection of the z-stack in a time course. (**B**) Effect of MLT+Ptero on NEDD9, Pro-PARP, and cleaved caspase-3 in HT29 and SW480 cells. Cell lysates were prepared and subjected to Western blotting for NEDD9, Pro-PARP, and cleaved caspase-3 in HT29 and SW480 cells. (**C**) Effect of NEDD9 overexpression on the cytotoxicity of MLT+Ptero in HT29, SW480, and HCT116 cells. Three colon cancer cells were transfected with the pcDNA-3.1 vector or NEDD9 overexpression plasmid for 48h and exposed to the indicated concentrations of MLT (1 mM) and/or Ptero (40 μM) for 24 h. Cytotoxicity by MLT+Ptero was evaluated by MTT assay in NEDD9 overexpressed HT29, SW480, and HCT116 cells.

Notably, MLT+Ptero completely reduced the intensity of GFP-NEDD9 fluorescence in HT29 cells compared to the untreated control 10 h after exposure to MLT+Ptero by Live Cell Microscopy (Figure 5A and Figure S1). Furthermore, NEDD9 overexpression reduced the cleavages of PARP and caspase 3 induced by MLT+Ptero in HT29 and SW480 cells (Figure 5B). Likewise, NEDD9 overexpression significantly reversed the cytotoxicity of MLT+Ptero in HT29, SW480, and HCT116 cells (Figure 5C).

### 2.6. Inhibition of miR-25-5p Rescued the Decreased Expression of NEDD in MLT+Ptero-Treated HT29 Cells

**Figure 6:**
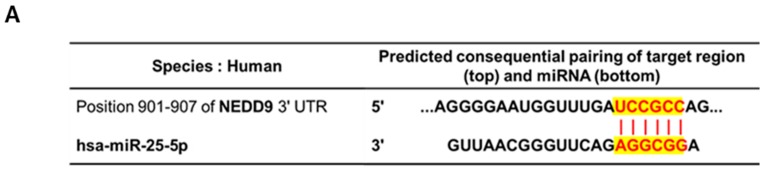

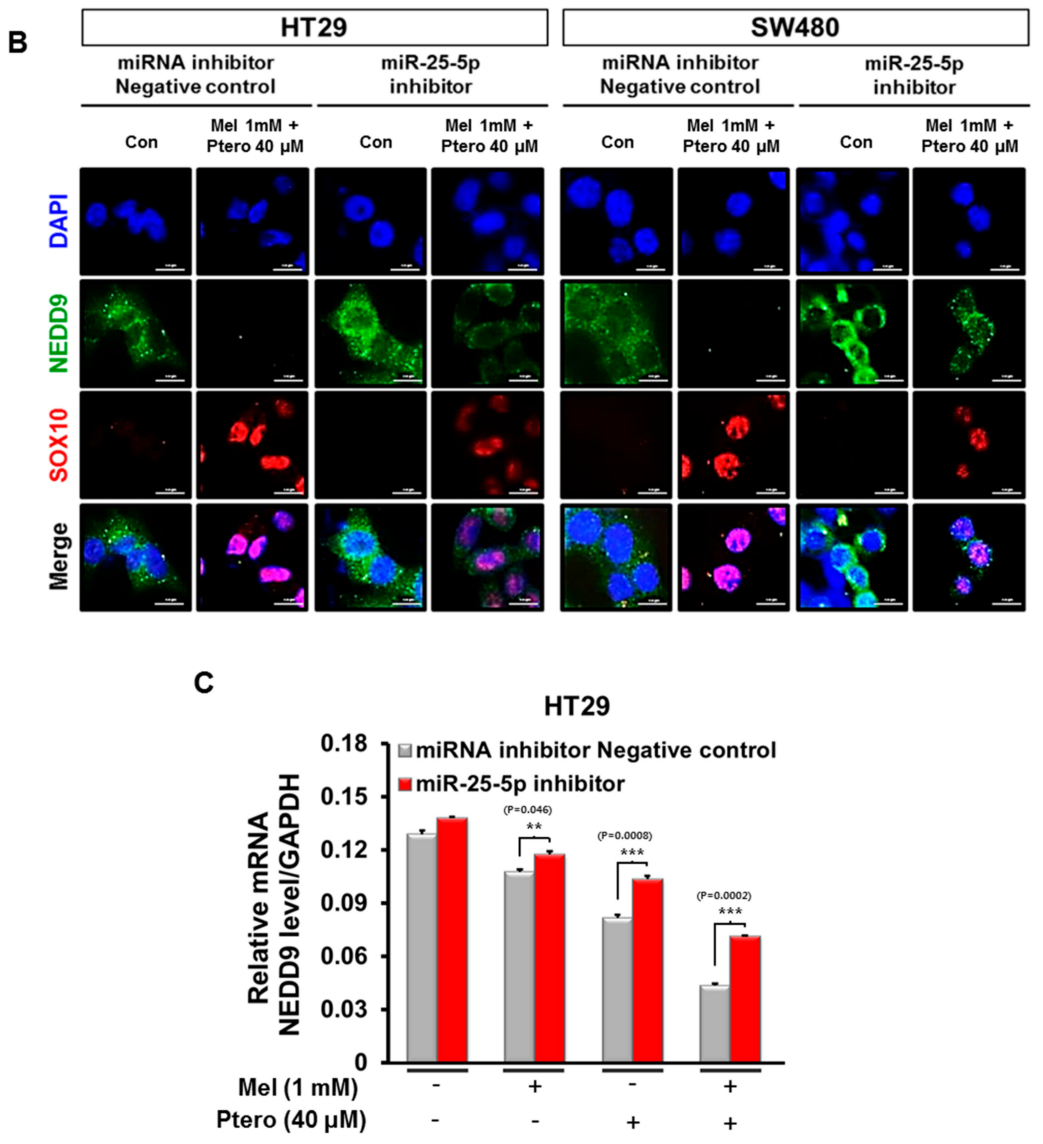
Critical role of miR-25-5p inhibitor in MLT+Ptero-treated colon cancer cells. (**A**) The binding sequences (red highlighted seed sequence) between 3′-UTR NEDD9 and hsa-miR-25-5p target genes by using miRWalk software. (**B**) Effect of miR-25-5p inhibitor on NEDD9 or SOX10 expression in MLT+Ptero-treated HT29 and SW480 cells. Expression levels of NEDD9 and SOX10 were analyzed by a Delta Vision Imaging System (Applied Precision). The nuclei were stained with DAPI (scale bar, 15 μm). (**C**) Effect of MLT+Ptero on NEDD9 in HT29 cells by qRT-PCR. After MLT+Ptero co-treatment for 24 h in HT29 cells, qRT-PCR was performed with total RNA isolated from HT29 cells. ** *p* < 0.01, *** *p* < 0.001 vs. miRNA inhibitor negative control. Data represent means ± SEM of triplicate samples.

Interestingly, miRWalk software as a stringent bioinformatics approach revealed that miR-25-5p directly bound to the 3′-Untranslated region (3′-UTR) (red highlighted sequence) of NEDD9 (Figure 6A). Here, inhibition of miR-25-5p reversed the decreased NEDD9 by MLT+Ptero in HT29 and SW480 cells by immufluorescence (Figure 6B). Consistently, inhibition of miR-25-5p rescued downregulation of NEDD by MLT+Ptero in HT29 cells by RT-PCR (Figure 6C).

### 2.7. Inhibition of miR-25-5p Reduced the Antiproliferative and Apoptotic Effects of MLT+Ptero in CRCs

**Figure 7:**
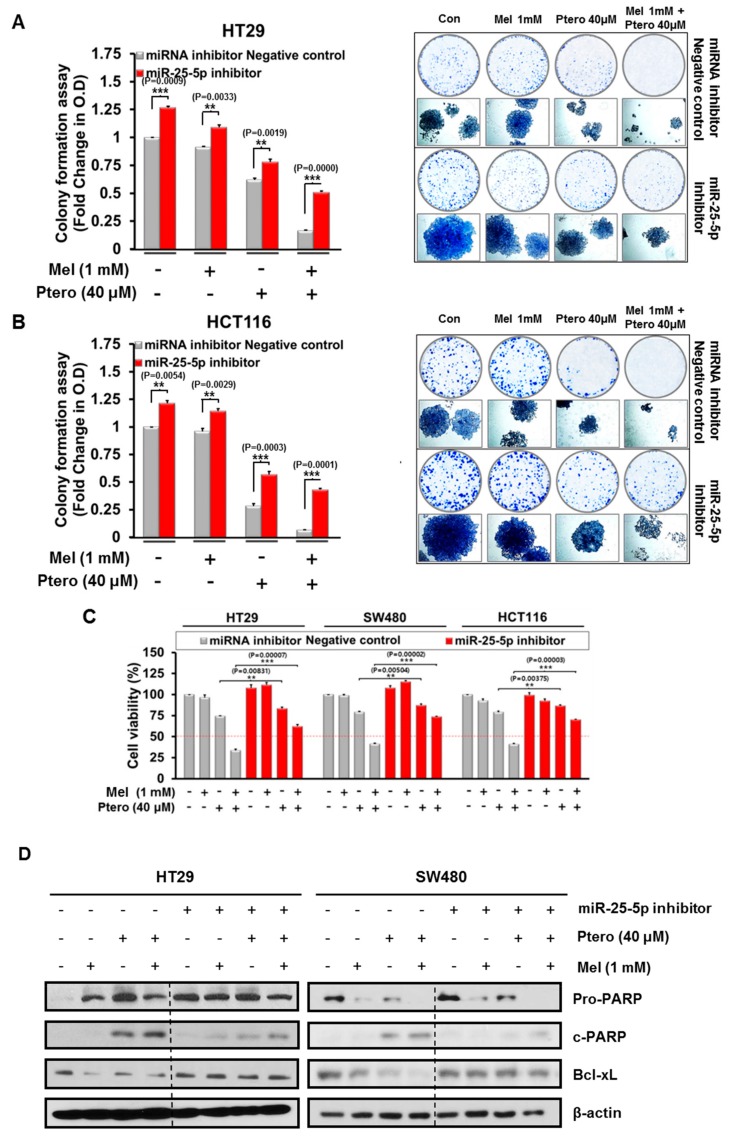
Effect of miR-25-5p inhibitor on colony formation, cell viability, and apoptosis-related proteins in MLT- and/or Ptero-treated colon cancer cells. Effect of miR-25-5p inhibitor on the colony formation of HT29 (A) and HCT116 (B) cells treated with MLT (1mM) and/or Ptero (40µM). Colony formation assay was conducted in HT29 and HCT116 cells transfected with miR-25-5p inhibitor plasmid compared to intact control cells for 2 weeks. (C) Effect of miR-25-5p inhibitor on the cytotoxicity of MLT+Ptero in HT29, SW480, and HCT116 cells. The miRNA inhibitor control and miR-25-5p inhibitor plasmids were transfected into three colon cancer cells for 48h and then exposed to MLT and/or Ptero for 24 h. Cell viability was determined by MTT assay. ** *p* < 0.01, *** *p* < 0.001 vs. miRNA inhibitor negative control. Data represent means ± SEM of triplicate samples. (D) Effect of miR-25-5p inhibitor on PARP cleavage and Bcl-xL in HT29 and SW480 cells treated with MLT and/or Ptero.

To validate the critical role of miR-25-5p in cytotoxicity and apoptosis by MLT+Ptero in CRCs, MTT assay, colony formation assay, and Western blotting were conducted in HT29, SW480, and HCT116 cells transfected with miR-25-5p inhibitor plasmid. Here, inhibition of miR-25-5p reversed the reduced number of colonies by MLT+Ptero in HT29 and HCT116 cells 2 week after MLT+Ptero co-treatment by colony formation assay (Figure 7A,B). Consistently, inhibition of miR-25-5p blocked cytotoxicity by MLT+Ptero in HT29, SW480, and HCT116 cells transfected with miR-25-5p inhibitor plasmid compared to MLT or Ptero alone by MTT assay (Figure 7C). Furthermore, Western blotting showed that suppression of miR-25-5p reduced PARP cleavage and the expression of Bcl-xL by MLT+Ptero in HT29 and SW480 cells (Figure 7D).

## 3. Discussion

In the current work, the underlying apoptotic mechanisms of MLT and Ptero were investigated in HT29, SW480, and HCT116 cells in association with miR-25-5p mediated NEDD9 signaling. Herein, MLT+Ptero revealed synergistic cytotoxicity with combination index below 1 compared to MLT or Ptero alone in three CRCs, implying the potent synergy of MLT+Ptero. Furthermore, MLT+Ptero decreased the numbers of colonies of HT29 and SW480 cells and decreased expression of proliferation marker Ki67 in HT29 cells, demonstrating the antiproliferative potential of MLT+Ptero co-treatment compared to MLT or Ptero alone. Also, MLT+Ptero increased the number of TUNEL-positive cells and ROS production, cleaved PARP, and caspase 3 as features of apoptosis [44] compared to MLT or Ptero alone in CRCs, indicating the synergistic apoptotic effect of MLT+Ptero.

It is well documented that SOX10 suppresses Wnt/β-catenin signaling and reduces epithelial-mesenchymal transition (EMT) migration and invasion of tumor cells, and enhances apoptosis as a tumor suppressor [45], whereas SOX9 enhances the growth of lung adenocarcinoma [46] with poor prognosis of patients with colorectal cancer [47]. Likewise, overexpression of NEDD9 suggests poor prognosis in patients with colorectal cancer [43] and promotes migration and progression of colon cancer cells through Wnt signaling [41], while SOX9 is known to mediate NEDD9 in melanomas. Here MLT+Ptero activated SOX10, reduced the expression of SOX9 and NEDD9 in HT29 and SW480 cells, and also completely attenuated fluorescence of NEDD9 overexpression in HT29 cells. Conversely, NEDD9 overexpression reversed cytotoxicity and cleavages of PARP and caspase 3 by MLT+Ptero, implying the pivotal role of NEDD9 inhibition in the apoptotic effect of MLT+Ptero.

Of note, microRNA microarray showed differential profile of microRNAs (upregulation: miR-25-5P, miR-711, miR-4725-3P, miR-542-5P, and miR-4484; downregulation: miR-4504, miR-195-3p, miR-668-3p, miR-3121-5p, and miR-5194) in MLT+Ptero-treated HT29 cells compared to MLT or Ptero alone. Among them, miR-25-5p was found most upregulated by MLT+Ptero in HT29 cells. Previous studies revealed that miR-25-5p acted as a tumor suppressor and PKCζ negative regulator in colon cancers [32,37], whereas miR-25 promotes proliferation of non-small cell lung cancer cells [33] and colorectal cancers as an oncogene [32]. Here, concentration-dependent upregulation of miR-25-5P was validated in MLT+Ptero-treated HT29 cells by qRT-PCR. Conversely, inhibition of miR-25-5p reversed the ability of MLT+Ptero to induce cytotoxicity, decrease colony formation, cleave PARP and attenuate the expression of Bcl-xL, reduce the green fluorescence of NEDD9, and increase SOX10 in CRCs transfected with miR-25-5p inhibitor plasmid, demonstrating the pivotal role of miR-25-5p in cytotoxic and antiproliferative effects of MLT+Ptero in CRCs.

Interestingly, miR-25-5p binds directly to the 3′-UTR sequence of NEDD9, and inhibition of miR-25-5p reversed the decreased expression of NEDD9 by MLT+Ptero by immufluorescence as well as rescued downregulation of NEDD at mRNA level by MLT+Ptero in HT29 cells by RT-PCR, strongly indicating close interaction between miR-25-5p and NEDD9 signaling, which should be confirmed by further study in vivo and in vitro in the near future.

## 4. Materials and Methods 

### 4.1. Cell Culture

Colon cancer cell lines including HCT116 (ATCC® CCL-247™), SW480 (ATCC® CCL-228™), HT29 (ATCC® HTB-38™), and CCD-18Co colon fibroblasts were purchased from American Type Culture Collection (ATCC, Manassas, VA, USA). The cells were cultured in Roswell Park Memorial Institute medium (RPMI) supplemented with 1% antibiotic (Welgene, Gyeongsan, Korea) and 10% fetal bovine serum (FBS).

### 4.2. Chemicals and Reagents

MLT, Ptero (trans-3,5-dimethoxy-4-hydroxystilbene;molecular weight: 256), phosphatase inhibitors, 3-(4,5-dimethylthiazol-2-yl)-2,5-diphenyltetrazolium bromide (MTT), and β-actin were bought from Sigma Aldrich (Sigma, St. Louis, MO, USA). Also, specific antibodies for PARP and NEDD9 were purchased from Cell signaling (Cell signaling, Beverly, MA, USA), SOX9, SOX10, and Bcl-xL from Santa Cruz (Santa Cruz Biotechnology, Dallas, CA, USA) for Western blotting. TUNEL kit and protease inhibitors were purchased from Roche (Roche Molecular Biochemicals, Mannheim, Germany) for TUNEL assay.

### 4.3. MTT Assay

Cell viability of Ptero and/or MLT was assessed in SW480, HT29, and HCT116 cells by MTT assay. Briefly, colon cancer cells (1 × 104 cells per well) were distributed onto 96-well microplate and exposed to various concentrations of MLT (0, 0.125, 0.25, 0.5, 0.75, 1, 1.25, 1.5 mM) and/or Ptero (0, 10, 20, 40, 60, 80, 100 μM) for one day. The cells were incubated with MTT (1 mg/mL) for 2 h, and then were exposed to MTT lysis solution overnight. Thereafter, optical density was measured by using a microplate reader (Molecular Devices Co., Silicon Valley, CA, USA) at 570 nm wavelength and the cell viability was calculated as a percentage of viable cells in Ptero- and/or MLT-treated group versus untreated control.

### 4.4. Colony Formation Assay

HCT116 and HT29 cells were seeded onto 6-well plates at a density of 103 cells per well in RPMI 1640 including 10% FBS for 24 h and then exposed to Ptero (40 μM) and/or MLT (1 mM) for 2 weeks. Then, colonies were stained with Diff-Quick solution (Sysmex, Kobe, Japan), washed once with PBS, and then the fields were photographed under a fluorescence microscope (AXIO observer A1, ZEISS, Oberkochen, Germany).

### 4.5. Observation of Apoptotic Morphological Features

Apoptotic morphology was observed one day after exposure to Ptero and/or MLT in HT29, SW480, and HCT116 cells under phase contrast microscope.

### 4.6. TUNEL Assay

The DeadEndTM TUNEL system kit was applied to detect cell death based on Roche’s instructions (Roche Molecular Biochemicals, Mannheim, Germany). Briefly, SW480 or HT29 cells exposed to Ptero and/or MLT for 24 h were washed with cold PBS and fixed with 4% paraformaldehyde for 30 min. Fixed cells in permeabilization solution (0.1% sodium citrate and 0.1%Triton X-100) were incubated with TUNEL assay mixture for 1 h. Then TUNEL-stained cells were visualized by a FLUOVIEW FV10i confocal microscopy (Olympus, Tokyo, Japan).

### 4.7. ROS Measurement

Hydrogen peroxide formation was analyzed using dichlorodihydrofluorescein diacetate (H_2_DCFDA) (Invitrogen, Carlsbad, CA, USA). HT29 or SW480 cells were treated with MLT (1 mM) and/or Ptero (40 μM) for 24h and then 10 μM DCFH-DA for 30 min at 37 °C. The fluorescence intensity was measured by fluorescence-activated cell sorting (FACS) Calibur.

### 4.8. Quantitative Real-Time Polymerase Chain Reaction (qRT-PCR)

Total RNAs from HT29 colon cells were isolated using the QIAzol (Invitrogen) and 1 µg of total RNA was applied for making cDNA by Superscript Reverse Transcriptase and then was amplified by Platinum Taq polymerase with Superscript One Step RT-PCR kit. Primers sequences used were synthesized by Bioneer (Daejeon, Korea). The primers for NEDD9 and GAPDH cDNA detection are as follows: NEDD9, 5′-CGCTGCCGAAATGAATAT-3′, and 5′-CCCTGTGTTCTGCTCTATGACG-3′; GAPDH, 5′-GCACCGTCAAGGCTGAGAAC-3′, and 5′-GGATCTCGCTCCTGGAAGATG-3′. PCR amplification steps were conducted based on Jung’s paper [6]. The amplified products were separated on 2% agarose gel and RT-qPCR was carried out with the LightCycler TM instrument (Roche Applied Sciences, Indianapolis, IN, USA).

### 4.9. Transfection Assay

The miR-25-5p inhibitor or NEDD9 overexpression plasmid and control vector (pDONR223, 200 nM) from Bioneer (Daejeon, Korea) were transfected into colon cancer cells using X-tremeGene transfection reagent (Roche Applied Biosystem, Basel, Switzerland). At 48 h after transfection, colon cancer cells were harvested after exposure to Ptero (40 µM) and/or MLT (1 mM) for one day for cell viability assay, Western blotting, and immunofluorescence.

### 4.10. Immunofluorescence Assay

Colon cancer cells treated by Ptero (40 µM) and/or MLT (1 mM) for 24 h were fixed with 4% formaldehyde and were permeabilized in 0.1% Triton X-100, according to the paper [13]. The fixed cells were washed with 1X PBS, blocked with 2% BSA in 1X PBS for 30 min at room temperature (RT), and incubated with the specific antibodies of NEDD9, SOX10, and Ki67 (1:1000; Abcam, Cambridge, UK) overnight at 4 °C. After washing, the cells were incubated with Alex Fluor 489 goat mouse-IgG antibody (Invitrogen) and Alexa Fluor 546 goat rabbit-IgG antibody (1:1000) for 1 h at RT. After washing twice, the nuclei of the cells were stained with 4,6-diamidino-2-phenylindole (DAPI; Sigma) and then were visualized under a FLUOVIEW FV10i confocal microscope (Olympus). Images of NEDD9- and SOX10-stained cells were taken by a Delta Vision imaging system (Applied Precision, Issaquah, WA, USA).

### 4.11. Western Blotting

HCT116, SW480, and HT29 cells were exposed to Ptero and/or MLT for 24 h and then were lysed in Radio-Immunoprecipitation Assay (RIPA) buffer (2 mM EDTA, 150 mM NaCl, 50 mM Tris-HCl, and 1% Triton X-100) containing protease inhibitors and phosphatase inhibitors. The protein samples were separated on 8% to 15% sodium dodecyl sulfate-polyacrylamide gels (SDS-PAGE) and were transferred to nitrocellulose membranes. The membranes were incubated with primary antibodies of PARP (Cell signaling, #9542), cleaved-caspase-3 (Cell signaling, #9661S), NEDD9 (Cell signaling, #4044S), SOX9 (Cell signaling, #82630S), SOX10 (Cell signaling, #69661S), Bcl-xL (Cell signaling, #2764), and β-actin (Sigma, A5316). These antibodies were diluted in 3% Bovine serum albumin (BSA) and in PBS-Tween20 (1:500–1:2000) at 4 °C overnight, washed with PBS-Tween20, and then incubated with HRP-conjugated secondary antibody (Santacruz, sc-516102, sc-2357) (1:2000). The protein expression was visualized by using Enhanced chemiluminescence (ECL) Western blotting detection reagent (GE Healthcare, Amersham, UK).

### 4.12. MicroRNA Microarray and Data Analysis

Based on the paper of [13], for control and test RNAs isolated from HT29 cells exposed to Ptero and/or MLT for 24 h, the syntheses of target miRNA probes and hybridization were conducted by using Agilent’s miRNA Labeling Reagent and Hybridization kit. Total RNAs (100 ng each) were dephosphorylated, denatured, and incubated for 10 min at 100 °C, ligated with pCp-Cy3 mononucleotide and purified with MicroBioSpin 6 columns (Bio-rad, Hercules, CA, USA). After purification, denatured labelled probes were transferred onto assembled Agilent Human miRNA Microarray (Human miRNA Microarray Release, AXBK) and hybridized for 20 h at 55 °C in an Agilent Hybridization oven (Agilent Technologies, Santa Clara, CA, USA). The hybridized microarrays were washed, and hybridized images were scanned using Agilent’s DNA microarray scanner and quantified with Feature Extraction Software (Agilent Technologies). All data were normalized (set measurements less than 0.01 to 0.01) and fold-changed probes (not less than 2.0-fold between test and control) were chosen and analyzed by using GeneSpringGX 7.3 (Agilent Technologies).

### 4.13. Statistical Analysis

The data values were expressed as means ± SD from at least three independent experiments. Student’s t-test was performed for two group comparison, while the one-way analysis of variance (ANOVA) followed by a Turkey post-hoc test was carried out for multi-group comparison using GraphPad Prism software (Version 5.0, San Diego, CA, USA). The statistical difference between groups was determined, only when p-value was less than 0.05.

## 5. Conclusions

MLT+Ptero exhibited significant cytotoxicity, suppressed the proliferative activity and Ki67 expression, increased TUNEL-positive cells and ROS production, cleaved PARP and caspase 3, attenuated the expression of Bcl-xL, NEDD9, and SOX9, and also activated SOX10 compared to MLT or Ptero alone in colon cancer cells. Also, MLT+Ptero induced differentially expressed microRNAs and also upregulated miR-25-5p at mRNA level in HT29 cells. Conversely, miR-25-5p inhibitor or NEDD9 overexpression reversed cytotoxicity, decreased colony formation, and PARP cleavage by MLT+Ptero in CRCs. Taken together, our findings provide new insights that co-treatment of MLT and Ptero synergistically enhanced apoptotic effect via miR-25-5p mediated NEDD9 signaling in CRCs as a potent therapeutic strategy for colorectal cancer prevention or treatment.

## Supplementary Materials

The following files are available online at https://www.mdpi.com/2072-6694/11/11/1684/s1: Table S1, heat map of microRNAs; Figure S1: movie of HT29 cells (control or MLT+Ptero).
